# Integrated Analysis of Transcriptome and Metabolome Reveals the Regulation of Chitooligosaccharide on Drought Tolerance in Sugarcane (*Saccharum* spp. Hybrid) under Drought Stress

**DOI:** 10.3390/ijms23179737

**Published:** 2022-08-27

**Authors:** Shan Yang, Na Chu, Hongkai Zhou, Jiashuo Li, Naijie Feng, Junbo Su, Zuhu Deng, Xuefeng Shen, Dianfeng Zheng

**Affiliations:** 1 College of Coastal Agricultural Sciences, South China Branch of National Saline-Alkali Tolerant Rice Technology Innovation Center, Guangdong Ocean University, Zhanjiang 524088, China; 2 National Engineering Research Center for Sugarcane, Fujian Agriculture and Forestry University, Fuzhou 350002, China; 3 South Subtropical Crops Research Institute, Chinese Academy of Tropical Agricultural Science, Zhanjiang 524091, China

**Keywords:** *Saccharum* spp. hybrid, drought stress, chitooligosaccharide, transcriptome, metabolome

## Abstract

Sugarcane (*Saccharum* spp. hybrid) is an important crop for sugar and biofuels, and often suffers from water shortages during growth. Currently, there is limited knowledge concerning the molecular mechanism involved in sugarcane response to drought stress (DS) and whether chitooligosaccharide could alleviate DS. Here, we carried out a combined transcriptome and metabolome of sugarcane in three different treatment groups: control group (CG), DS group, and DS + chitooligosaccharide group (COS). A total of 12,275 (6404 up-regulated and 5871 down-regulated) differentially expressed genes (DEGs) were identified when comparing the CG and DS transcriptomes (T_CG/DS), and 2525 (1261 up-regulated and 1264 down-regulated) DEGs were identified in comparing the DS and COS transcriptomes (T_DS/COS). GO and KEGG analysis showed that DEGs associated with photosynthesis were significantly enriched and had down-regulated expression. For T_DS/COS, photosynthesis DEGs were also significantly enriched but had up-regulated expression. Together, these results indicate that DS of sugarcane has a significantly negative influence on photosynthesis, and that COS can alleviate these negative effects. In metabolome analysis, lipids, others, amino acids and derivatives and alkaloids were the main significantly different metabolites (SDMs) observed in sugarcane response to DS, and COS treatment reduced the content of these metabolites. KEGG analysis of the metabolome showed that 2-oxocarboxylic acid metabolism, ABC transporters, biosynthesis of amino acids, glucosinolate biosynthesis and valine, leucine and isoleucine biosynthesis were the top-5 KEGG enriched pathways when comparing the CG and DS metabolome (M_CG/DS). Comparing DS with COS (M_DS/COS) showed that purine metabolism and phenylalanine metabolism were enriched. Combined transcriptome and metabolome analysis revealed that pyruvate and phenylalanine metabolism were KEGG-enriched pathways for CG/DS and DS/COS, respectively. For pyruvate metabolism, 87 DEGs (47 up-regulated and 40 down-regulated) and five SDMs (1 up-regulated and 4 down-regulated) were enriched. Pyruvate was closely related with 14 DEGs (|r| > 0.99) after Pearson’s correlation analysis, and only 1 DEG (*Sspon.02G0043670-1B*) was positively correlated. For phenylalanine metabolism, 13 DEGs (7 up-regulated and 6 down-regulated) and 6 SDMs (1 up-regulated and 5 down-regulated) were identified. Five PAL genes were closely related with 6 SDMs through Pearson’s correlation analysis, and the *novel.31257* gene had significantly up-regulated expression. Collectively, our results showed that DS has significant adverse effects on the physiology, transcriptome, and metabolome of sugarcane, particularly genes involved in photosynthesis. We further show that COS treatment can alleviate these negative effects.

## 1. Introduction

During growth and development, plants are affected by biotic and abiotic stresses that can reduce yield and quality [1]. Abiotic stresses include high temperature stress, low temperature stress, heavy metal stress, salt stress and drought stress (DS). DS is a major environmental stress that can threaten plant survival and crop production [2]. DS hinders photosynthesis and normal metabolic function to affect plant growth and development [3]. Sugarcane is a C4 plant, belonging to the family *Gramineae*, genus *Saccharum*. Growth of sugarcane requires substantial amounts of water, especially during the jointing stage when growth is particularly vigorous [4]. The jointing stage is also the period that has the greatest influence on final yield and when sugarcane is most sensitive to DS [5]. Therefore, study of physiological, metabolic, and transcriptional regulation mechanisms of sugarcane at jointing stage under DS is needed to preserve yield as the frequency of drought increases worldwide.

Plant growth regulators (PGRs) are synthetic compounds that at low concentration can influence plant growth and development [6]. Hormones such as indole-3-acetic acid (IAA), gibberellin (GA), abscisic acid (ABA), ethylene (ETH), cytokinin (CK), brassinosteroids (BRs), salicylic acid (SA), and jasmonic acid (JA) are common PGRs [7]. These compounds are important regulators of plant growth and mediate responses to both biotic and abiotic stresses. Cytokinin-auxin antagonistic interactions that control root development are well-characterized [8]. By regulating the content of auxin and abscisic acid during seed germination, indole-3-acetate beta-glucosyltransferase (*OsIAGLU*) can affect expression of the downstream ABA signaling factor (*OsABIs*) and determine the vigor level of rice seeds [9]. In *Arabidopsis thaliana*, glucose (Glc) functions as a hormone-like signaling molecule that modulates plant growth and development. Exogenous application of 1, 3 and 5% Glc represses primary root growth by shortening the meristematic zone of roots [10]. Chitosan is a main byproduct of processing of crab and shrimp shells as well as fish scales, and is used in foliar applications for various agricultural crops [11]. Chitooligosaccharide is a degradation product of chitosan that has a polymerization degree ranging between 2 and 20 and is not poisonous. Compared with chitosan, chitooligosaccharide has good water solubility, moisture absorption, and moisture retention, as well as antibacterial properties and special physiological activities. Application of oligo-chitosan (50–75 mg/L) as a foliar spray for potato plants under DS reduced the membrane stability index and malondialdehyde, whereas antioxidase activities and chlorophyll, proline, and total sugars amounts were enhanced considerably [12]. Thus, oligo-chitosan can effectively improve the drought tolerance of potato, suggesting that it may be able to alleviate biological stress in other plants.

Plants respond to DS by a series of changes in gene expression as well as physiological, biochemical, and metabolic activity. Transcriptomics is the study of gene expression at the RNA level using high-throughput sequencing. RNA-seq of sugarcane treated with PEG6000 (20%) at seedling stage to induce DS showed differential expression of *AP2/ERF* transcription factors. In particular, expression of 12 *SsAP2/ERF* genes was induced in response to DS [13]. Examination of leaf transcriptomes using Illumina NextSeq sequencing with GO analysis of two sugarcane genotypes exposed to DS showed that most of the enriched genes were associated with peroxidase activity, response to oxidative stress, and response to stress, indicating that DS can produce peroxidation and cellular damage [14]. The mechanism of DS tolerance in plants is complex and involves intricate regulatory networks. Thus, understanding the relationship between genes and downstream metabolites is important to combat negative effects of DS. Metabolomics is a robust approach for plant biologists to understand complex metabolic responses to various abiotic pressures [15]. Metabolites can be examined qualitatively and quantitatively using widely targeted metabolome technology. Metabolome profiling of cultivars IR64 (drought sensitive) and Apo (drought tolerant) exposed to different water conditions (50% field capacity and 100% field capacity) showed that expression of genes associated with the phenylpropanoid pathway, carbohydrate metabolism, and sucrose transporters were up-regulated in both cultivars, but accumulation of amino acids was lower in Apo than IR64 [16]. Furthermore, key metabolites including sucrose, putrescine, glutamate, serine, and myo-inositol related to axillary bud outgrowth were revealed through metabolite profiling [17]. Accumulation of metabolites is closely connected to gene expression regulation. Combined transcriptome and metabolome analysis revealed that cyanidin, cyanidin 6’-malonylglucoside, cyanidin O-glucoside, and peonidin O-glucoside are the main components responsible for sugarcane rind color, and 50 unigenes belonging to 15 enzyme families were identified as putative genes involved in anthocyanin biosynthesis in sugarcane rind [18]. These studies demonstrate the potential for in-depth study of gene–gene, metabolite–metabolite, and gene-metabolite regulatory networks that can be carried out using transcriptome and metabolome analysis.

Previous studies on sugarcane response to DS were conducted at a transcriptome or metabolome level without integrative analysis of multi-omics approaches. In this study, we aimed to reveal the molecular regulatory network of genes and metabolites related to DS response and chitooligosaccharide regulation of drought tolerance of sugarcane based on comprehensive analysis of the transcriptome and metabolome. The findings of the present study could enrich our understanding of sugarcane response to DS and provide a theoretical framework for breeding and cultivation strategies that enhance drought tolerance of sugarcane.

## 2. Results

### 2.1. Effect Analysis of Roc22 Phenotype and Physiology under Different Treatments

**Figure 1:**
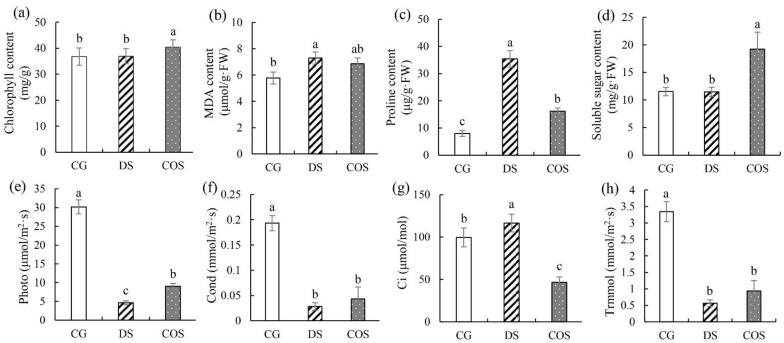
Physiological and biochemical parameters under different treatments. (**a**) Variation diagram of chlorophyll content. (**b**) Variation diagram of malondialdehyde (MDA) content. (**c**) Variation diagram of proline content. (**d**) Variation diagram of soluble sugar content. (**e**) Variation diagram of Photo. (**f**) Variation diagram of Cond. (**g**) Variation diagram of Ci. (**h**) Variation diagram of Trmmol. Photo, Cond, Ci, and Trmmol indicate net photosynthesis, stomatal conductance, intercellular carbon dioxide concentration, and transpiration rate, respectively. Error bars represent standard deviation of three samples. Different letters on bars indicate statistically significant differences between treatments (*p* < 0.05).

Compared to the control group (CG), the phenotypes were significantly different for drought stress (DS) plants after 10 d DS, including leaf rolling, reduced biomass, and shorter plant height (Figure S1). Different indexes were also seen for the 3 treatments (CG, DS, COS; Figure 1). The chlorophyll content and soluble sugar content were similar between the CG group and DS group but were significantly increased in the drought stress + chitooligosaccharide (COS) group (Figure 1). Similar trends were seen for variations in malondialdehyde (MDA) content, proline content, and Ci, in that the DS group was higher than the CG group, and the COS group was less than the DS group (Figure 1). Furthermore, similar variation in Photo, Cond and Trmmol were seen, with the CG group having the highest levels and DS having the lowest (Figure 1). These results showed that DS inhibited photosynthesis and physiological activity of plant and that chitooligosaccharide could alleviate the effects of DS to some extent.

### 2.2. Transcriptome Analysis

The raw data for 9 transcriptome libraries were subjected to quality control by filtering and cleaning the data and checking the sequencing error rate and GC content distribution prior to subsequent analysis. The Q20 and Q30 of the 9 libraries were all above 96.68% and 91.11%, respectively, and the GC content was between 51.24% and 55.74%, indicating that the data had high accuracy and could meet the requirements of the bioinformatics analysis carried out for this study (Table S1). The clean reads were mapped to the *Saccharum spontaneum* reference genome using HISA2, and the mapping efficiency of the 9 libraries was >84.82% (Table S1). To validate the RNA-seq data, we selected 10 differentially expressed genes (DEGs) for RT-qPCR analysis of samples exposed to different DS treatments. The results of RT-qPCR analysis were consistent with those for RNA-seq, suggesting that the RNA-seq data was accurate (Figure S2). We identified 10 DEGs that are involved in pyruvate metabolism and phenylalanine metabolism. 

The DEGs were identified using DESeq2 (|log_2_fold change| ≥ 1 and FDR < 0.05). A total of 12,275 DEGs were identified when comparing the transcriptomes for the CG and DS groups (CG vs. DS; T_CG/DS), of which 6404 DEGs had up-regulated expression and 5871 DEGs had down-regulated expression, indicating that DS could induce differential gene expression in sugarcane (Figure S3). A total of 2525 DEGs were identified when comparing the DS and COS transcriptomes (DS vs. COS; T_DS/COS), of which 1261 were up-regulated and 1264 were down-regulated, suggesting that chitooligosaccharide treatment could affect gene expression of sugarcane under DS (Figure S3). 

**Figure 2:**
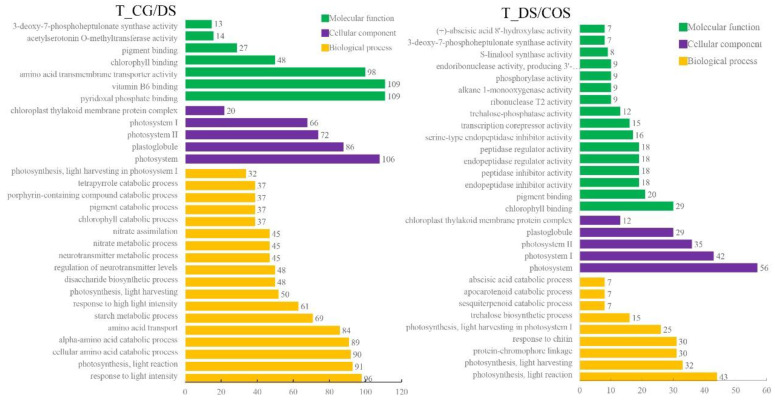
Top-30 GO pathway enrichment of DEGs in the DS and COS treatment groups compared to the control group (CG/DS; left panel) and to each other (DS/COS; right panel). Numbers beside the columns indicate the number of DEGs in that pathway. Green, purple, and yellow bars represent molecular function (MF), cellular component (CC), and biological process (BP), respectively.

GO enrichment analysis indicated that 3541 DEGs were enriched in the 562 GO terms for T_CG/DS (*p* < 0.005), and, of these, 322 GO terms belonged to biological process (BP), 25 belonged to cellular component (CC), and 215 belonged to molecular function (MF) (Table S2). The top-3 enriched terms for BP were response to light intensity, photosynthesis-light reaction, and cellular amino acid catabolic process (Figure 2). The top-3 enriched terms for CC were photosystem, plastoglobuli, and photosystem II (Figure 2). For MF, top-3 enriched terms were pyridoxal phosphate binding, vitamin B6 binding, and amino acid transmembrane transporter activity (Figure 2). Therefore, the main DEGs under DS were genes related to photosynthesis, suggesting that DS had a substantial effect on photosynthetic activity of sugarcane. For T_DS/COS, 637 DEGs were enriched in 257 GO terms, of which 149 belonged to BP, 16 belonged to CC, and 93 belonged to MF (Table S3). For BP, the top-3 enriched terms were photosynthesis-light reaction, photosynthesis-light harvesting, and protein-chromophore linkage, and for CC the top-3 were photosystem, photosystem I, and photosystem II. For MF, the top-3 terms were chlorophyll binding, pigment binding, and endopeptidase inhibitor activity, indicating that chitooligosaccharide also regulated photosynthetic activity of sugarcane under DS (Figure 2).

**Figure 3:**
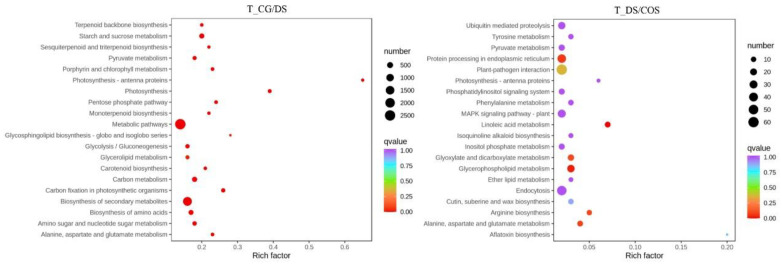
Top-20 KEGG pathway enrichment of DEGs in the DS and COS treatment groups compared to the control group (CG/DS; left panel) and to each other (DS/COS; right panel). The size of the circles corresponds to the number of DEGs and are color-coded according to q-value. The x-axis shows the enrichment factor value.

KEGG pathway enrichment analysis of DEGs showed that 62 and 23 KEGG pathways were significantly enriched for T_CG/DS and T_DS/COS (*p* < 0.05), respectively (Tables S4 and S5). In T_CG/DS, the top-5 enriched pathways were starch and sucrose metabolism, photosynthesis, photosynthesis-antenna proteins, carbon fixation in photosynthetic organisms, and carbon metabolism, which were represented by 292 (136 up-regulated and 156 down-regulated), 74 (13 up-regulated and 61 down-regulated), 39 (1 up-regulated and 38 down-regulated), 101 (30 up-regulated and 71 down-regulated), and 245 (108 up-regulated and 137 down-regulated) DEGs, respectively, suggesting that genes related to photosynthesis had down-regulated expression in response to DS (Figure 3). For the T_DS/COS group, the top-5 enriched pathways were photosynthesis-antenna proteins (25 up-regulated DEGs), photosynthesis (26 up-regulated and 3 down-regulated DEGs), starch, and sucrose metabolism (31 up-regulated and 37 down-regulated DEGs), protein processing in endoplasmic reticulum (56 up-regulated and 8 down-regulated DEGs), and porphyrin and chlorophyll metabolism (7 up-regulated and 13 down-regulated DEGs) (Figure 3). Compared with T_CG/DS, most DEGs in T_DS/COS were up-regulated, demonstrating that chitooligosaccharide treatment could alleviate the negative effects of DS in sugarcane.

### 2.3. Metabolome Analysis

LC-MS/MS was used to analyze ROC22 metabolites under different treatment conditions to investigate variations in the metabolomic profiles in response to DS and the regulatory effect of chitooligosaccharide. Through screening of metabolites that had significantly different levels, a total of 269 significantly different metabolites (SDMs; 216 up-regulated and 53 down-regulated) were found when comparing the metabolites of control plants with plants exposed to DS (M_CG/DS). For M_DS/COS, 123 SDMs (33 up-regulated and 90 down-regulated) were found (Figure S4). Heat map analysis of the metabolites showed significant differences among the different treatments. Lipids, others (sugars and alcohols), amino acids and derivatives, and alkaloids were up-regulated in the M_CG/DS, but down-regulated in the M_DS/COS (Figure S5). Therefore, SDMs were induced and up-regulated when sugarcane was subjected to DS, and chitooligosaccharide treatment could alleviate DS and down-regulate SDMs in sugarcane. There were 38, 33, 52, and 35 SDMs belonging to lipids, others, amino acids and derivatives, and alkaloids in the M_CG/DS, and accounted for 17.59%, 15.28%, 24.07%, and 16.20% of the 216 up-regulated SDMs, respectively (Tables S6 and S7). There were 15, 11, 11 and 16 SDMs that belonged to lipids, others, amino acids and derivatives, and alkaloids in the M_DS/COS, accounting for 16.67%, 12.22%, 12.22%, and 17.78% of 90 down-regulated SDMs, respectively (Tables S6 and S8). As such, lipids, others, amino acids and derivatives, and alkaloids were the main SDMs in response to DS in sugarcane and chitooligosaccharide treatment alleviated DS and reduced the content of these metabolites.

**Figure 4:**
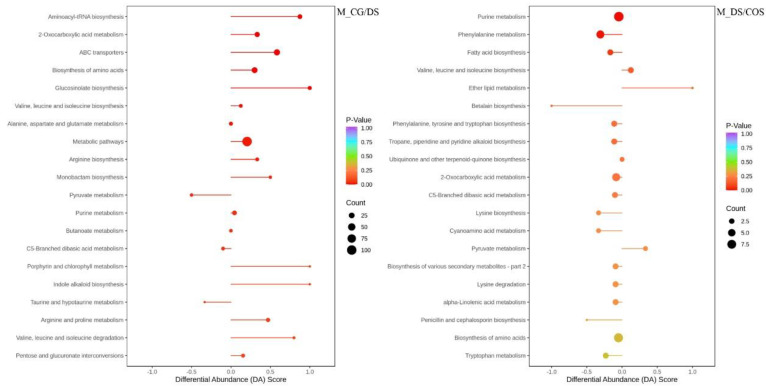
Top-20 KEGG pathway enrichment of SDMs in the DS and COS treatment groups compared to the control group (CG/DS; left panel) and to each other (DS/COS; right panel) as a function of differential abundance (DA) score. The size of the circles corresponds to number of DEGs and are color-coded according to *p*-value.

According to the KEGG pathway enrichment analysis, 13 and 2 KEGG pathways were significantly enriched in M_CG/DS and M_DS/COS, respectively (*p* < 0.05; Tables S9 and S10). For M_CG/DS, the top-5 KEGG enriched pathways were 2-oxocarboxylic acid metabolism, ABC transporters, biosynthesis of amino acids, glucosinolate biosynthesis and valine, leucine and isoleucine biosynthesis, containing 18 (13 up-regulated and 5 down regulated), 30 (29 up-regulated and 1 down regulated), 26 (19 up-regulated and 7 down regulated), 8 (8 up-regulated), and 7 (4 up-regulated and 3 down regulated) SDMs, respectively (Figure 4 and Table S9). Most of these KEGG enriched pathways were up-regulated under DS in sugarcane (Figure 4). For M_DS/COS, there were 2 KEGG enriched pathways, namely purine metabolism and phenylalanine metabolism, containing 9 (4 up-regulated and 5 down regulated) and 6 SDMs (1 up-regulated and 5 down-regulated), respectively (Figure 4 and Table S10). Thus, chitooligosaccharide affected SDMs associated with purine metabolism and phenylalanine metabolism that were down-regulated under DS.

### 2.4. Combined Transcriptome and Metabolome Analysis

**Figure 5:**
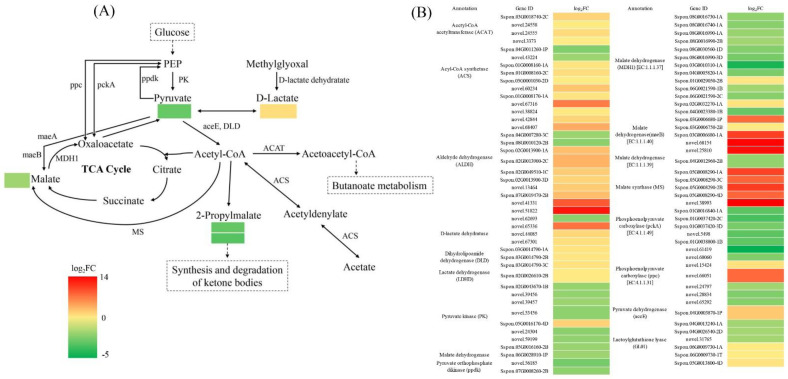
Analysis of DEGs and SDMs in the pyruvate metabolic pathway for the CG/DS group. (**A**) Metabolic pathways examined in KEGG analysis. (**B**) Genes involved in KEGG metabolic pathways. Color-coding in A and B corresponds to observed fold-change.

**Figure 6:**
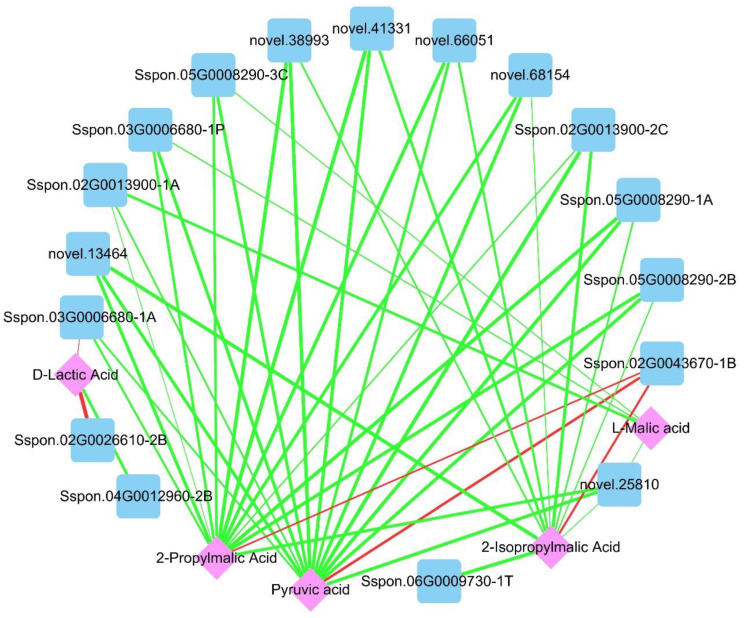
Gene-metabolite network of pyruvate metabolism for the CG/DS comparison (|r| ≥ 0.990). Blue squares and pink diamonds represent genes and metabolites, respectively. Green lines indicate negative correlations and red lines are positive correlations. The line thickness represents the magnitude of the correlation coefficient, which ranged from 0.990 to 0.998.

To further explore the effects of DS on genes and metabolites in sugarcane, we carried out a combined transcriptome and metabolome analysis. A Venn diagram analysis showed 8 common KEGG enriched pathways between T_CG/DS and M_CG/DS, namely glucosinolate biosynthesis, pyruvate metabolism, alanine, aspartate and glutamate metabolism, arginine biosynthesis, metabolic pathways, 2-oxocarboxylic acid metabolism, butanoate metabolism, and biosynthesis of amino acids (Figure S6, Tables S4 and S9). Pyruvate is an intermediate that connects photosynthesis and respiration, and also serves as a hub for the mutual conversion of sugars, fats, and amino acids. Based on this important role and its positive response to DS in sugarcane, the pyruvate metabolism was selected for further analysis. For T_CG/DS and M_CG/DS, 87 DEGs (47 up-regulated and 40 down-regulated) and 5 SDMs (1 up-regulated and 4 down-regulated) were enriched for pyruvate metabolism, respectively (Figure 5). These DEGs were annotated and classified into 17 types, of which aldehyde dehydrogenase (ALDH), malate dehydrogenase (MDH1), and pyruvate kinase (PK) were the top-3 and contained 15 (12 up-regulated and 3 down-regulated), 13 (2 up-regulated and 11 down-regulated), and 8 (1 up-regulated and 7 down-regulated) DEGs, respectively (Figure 5). The 5 SDMs were D-lactic acid, L-malic acid, pyruvic acid, 2-propylmalic acid and 2-isopropylmalic acid, of which D-lactic acid was the only up-regulated SDM (Figure 5). To understand the relationship between differential genes and differential metabolites, we produced a network diagram based on Pearson’s correlation analysis. Pearson’s correlation analysis results showed that 5 SDMs were closely related with 59 DEGs (*p* < 0.01 and |r| > 0.900; Table S11). Pyruvate was closely related to 14 DEGs (|r| ≥ 0.990), of which only 1 DEG (*Sspon.02G0043670-1B*) had a positive correlation with pyruvate (Figure 6). For L-malic acid, there were 4 DEGs that had a significant negative correlation (|r| ≥ 0.990), namely *novel.25810*, *Sspon.05G0008290-3C*, *Sspon.03G0006680-1P*, and *Sspon.02G0013900-1A* (Figure 6). D-lactic acid had a significant correlation with 3 DEGs (|r| ≥ 0.990), namely *Sspon.02G0026610-2B* (positive correlation), *Sspon.03G0006680-1A* (positive correlation), and *Sspon.04G0012960-2B* (negative correlation) (Figure 6). These DEGs were significantly correlated with important SDMs (pyruvate, L-malic acid and D-lactic acid) in pyruvate metabolism, and could be candidate drought tolerance genes for future studies on the mechanisms of drought tolerance in sugarcane.

**Figure 7:**
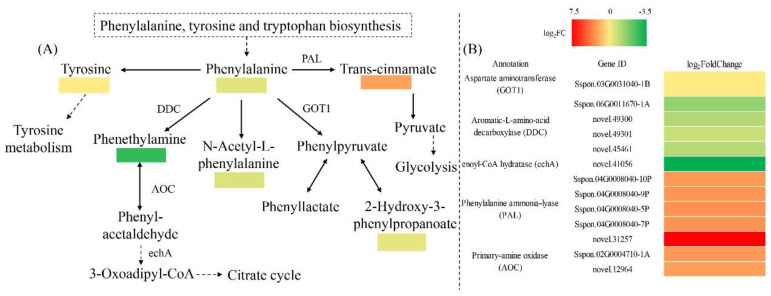
Analysis of DEGs and SDMs in the phenylalanine metabolic pathway when comparing the DS and COS groups. (**A**) Enriched SDMs and (**B**) DEGs of the phenylalanine metabolic pathway. |log_2_FC| denotes |log_2_fold change|.

**Figure 8:**
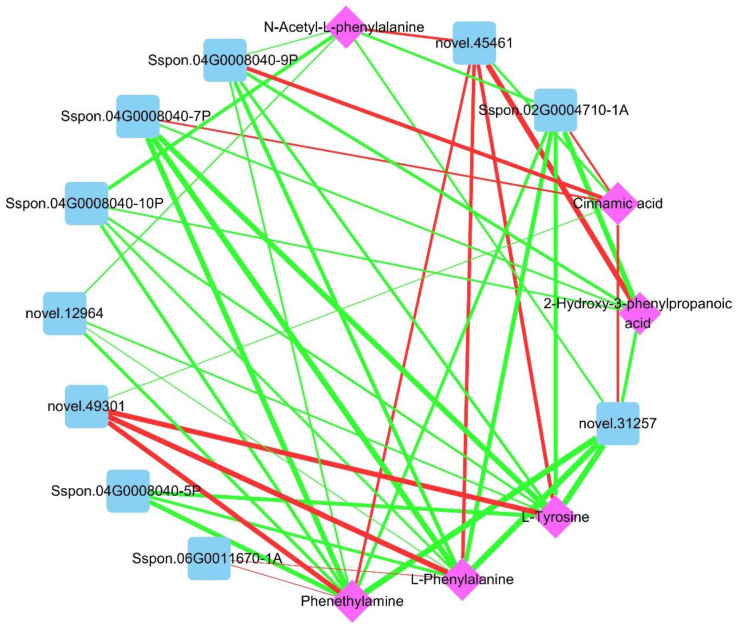
Gene–metabolite network graph of phenylalanine metabolism when comparing the DS and COS groups. Blue squares and pink diamonds represent genes and metabolites, respectively. Green lines indicate negative correlations and red lines are positive correlations. The line thickness represents the magnitude of the correlation coefficient, which ranged from 0.919 to 0.998.

We next performed an integrative analysis of the transcriptome and metabolome to examine the regulatory effects of chitooligosaccharide on sugarcane under DS. Phenylalanine metabolism was the only KEGG enriched pathway shared by T_DS/COS and M_DS/COS (Tables S5 and S10). In phenylalanine metabolism, 13 DEGs (7 up-regulated and 6 down-regulated) and 6 SDMs (1 up-regulated and 5 down-regulated) were seen for T_DS/COS and M_DS/COS, respectively (Figure 7). Among the 13 DEGs, 5 were phenylalanine ammonia-lyase (PAL, up-regulated), 4 were aromatic-L-amino-acid decarboxylase (DDC, down-regulated), 2 were primary-amine oxidase (AOC, up-regulated), and 1 each for aspartate aminotransferase (GOT1, down-regulated) and enoyl-CoA hydratase (echA, down-regulated) (Figure 7). The 6 SDMs were L-phenylalanine, phenethylamine, cinnamic acid, N-acetyl-L-phenylalanine, 2-hydroxy-3-phenylpropanoic acid, and L-tyrosine, of which cinnamic acid was the only up-regulated SDM (Figure 7). Therefore, chitooligosaccharide treatment positively regulated phenylalanine metabolism under DS in sugarcane, resulting in reduced phenylalanine content and increased amounts of cinnamic acid, that together allowed synthesis of more downstream phenylalanine secondary metabolites that engage in metabolic activities in response to DS. The gene–metabolite network graph analysis showed that L-phenylalanine, L-tyrosine, and cinnamic acid were indeed significantly correlated with 10 DEGs (3 positive and 7 negative relationship), 9 DEGs (2 positive and 7 negative relationship), and 6 DEGs (4 positive and 2 negative relationship), respectively (Figure 8 and Table S12). L-phenylalanine was strongly associated with *novel.49301* (r = 0.988), *novel.45461* (r = 0.960), *Sspon.04G0008040-9P* (r = −0.966), *Sspon.02G0004710-1A* (r = −0.975), *Sspon.04G0008040-7P* (r = −0.994), and *novel.31257* (r = −0.998) in the DS/COS group (Figure 8 and Table S12). L-tyrosine was closely related to *novel.49301* (r = 0.985), *novel.45461* (r = 0.963), *Sspon.04G0008040-5P* (r = −0.965), *Sspon.02G0004710-1A* (r = −0.969), *Sspon.04G0008040-7P* (r = −0.988), and *novel.31257* (r = −0.997) (Figure 8 and Table S12). Cinnamic acid was closely related to the DEGs, namely *Sspon.04G0008040-9P* (r = 0.967), *novel.31257* (r = 0.946), and *novel.45461* (r = −0.945) (Figure 8 and Table S12). These DEGs and SDMs were closely related to regulation by chitooligosaccharide under DS.

## 3. Discussion

The increasing frequency of water shortages and the emergence of extreme weather has seriously affected the quality and yield of many crops [19]. Many crops are now affected by drought stress (DS) during growth. Responses to DS by crops involve multiple processes, including perception, signal transduction, osmoprotection, transcription, translation, protein modification, and metabolic regulation [20]. In sugarcane, plant height, plant diameter, total leaf area, and dry matter accumulation were significantly reduced by DS [21]. In this study, we saw similar responses to DS, particularly reduced plant height and withered leaves, which are adaptive traits to protect against water loss. Chlorophyll content, net photosynthesis (Photo), stomatal conductance (Cond), and transpiration rate (Trmmol) of sugarcane plants were also reduced under DS, but the intercellular carbon dioxide concentration (Ci) increased, indicating that DS had negative influence on sugarcane photosynthesis. Six sugarcane varieties were previously shown to suffer from drought stress in the tillering stage, as well as stages when intense growth and ripening occur, and the photosynthetic apparatus was particularly and severely affected by drought, as evidenced by reduced photosynthetic rate and chlorophyll content [22]. Meanwhile, DS induces peroxidation and osmotic stress in plants. In this study, the MDA content, proline content, and soluble sugar content were indeed significantly increased by DS. Abbas et al. found that drought tolerant sugarcane genotypes had higher antioxidant activities and stronger capacity for osmotic adjustment [23]. Sugarcane does synthesize osmoprotectants to regulate osmotic pressure in response to DS, which is an adaptation to drought. 

Chitosan is highly protective against the most dangerous diseases and pathogens for crops, and also can improve yield and chlorophyll content, as well as some plant growth parameters [11]. In this study, plants exposed to DS and treated with the chitooligosaccharide had higher chlorophyll content and were greener, and soluble sugar content compared to DS plants (Figure S1 and Figure 1). Photo, Cond, and Trmmol were also higher in the COS group, whereas the MDA content, proline content, and Ci were lower relative to DS, suggesting that exogenous application of chitooligosaccharide may enhance photosynthesis and osmotic adjustment in sugarcane exposed to DS. In rice, exogenous application of chitosan (50–190 kDa) promoted root growth under DS, with higher relative content of water and photosynthetic pigments, whereas lower concentrations of chitosan had higher efficacy [24]. Chitosan treatments also alleviated the effects caused by DS in barley, as evidenced by the decreases in electrolyte leakage and levels of both MDA and hydrogen peroxide (H_2_O_2_) that corresponded to increases in activities of the antioxidant enzymes superoxide dismutase (SOD), catalase (CAT), ascorbate peroxidase (APX), and guaiacol peroxidase (GPX) activity [25]. Therefore, chitooligosaccharide (≤3200 Da) had a higher efficacy in alleviating the negative effects of DS in this study. However, the molecular regulatory network by which these alleviating effects of chitooligosaccharide are mediated in sugarcane requires further investigation.

Transcriptome analysis can reveal a series of genes that exhibit differential expression in response to DS. In this study, a total of 12,275 DEGs (6404 up-regulated and 5871 down-regulated) were identified in the CG/DS comparison and 2525 DEGs (1261 up-regulated and 1264 down-regulated) were identified in the DS/COS comparison, indicating that there were significant differences in gene expression among the three experimental groups. GO enrichment analysis indicated that GO terms for the DEGs were mainly enriched in photosynthesis, both for the CG/DS and DS/COS comparisons. Moreover, KEGG analysis showed that starch and sucrose metabolism, photosynthesis, and photosynthesis-antenna proteins were among the top-5 enriched pathways for both T_CG/DS and T_DS/COS. Interestingly, for T_DS/COS, differential levels were seen for 68 (31 up-regulated and 37 down-regulated), 29 (26 up-regulated and 3 down-regulated), and 25 (25 up-regulated) DEGs related to starch and sucrose metabolism, photosynthesis, and photosynthesis-antenna proteins, respectively. Therefore, DS had negative effects on photosynthesis in sugarcane that could be alleviated by chitooligosaccharide treatment. Previous studies showed that water stress affects not only light reactions, but also assimilation efficiency of the dark reactions, thereby reducing the contents of photosynthetic products [26,27]. 

Differential metabolites are induced and accumulated by biotic and abiotic stress in a manner that is most closely related to phenotype [28]. Zhao et al. found that metabolites of two rice cultivars were temporally, tissue-specifically, and genotype-dependently regulated under salt stress, and sugars and amino acids increased significantly in the leaves and roots [29]. Vital et al. found that soluble sugar, secondary metabolite production, and activation of ROS eliminating processes are involved in drought tolerance in sugarcane [5]. In this study, we found similar results. We found 269 SDMs (216 up-regulated SDMs and 53 down-regulated SDMs) for M_CG/DS and 123 SDMs (33 up-regulated SDMs and 90 down-regulated SDMs) for M_DS/COS, indicating that SDMs were induced and up-regulated by DS, and that chitooligosaccharide could alleviate DS and reduce the SDM content in sugarcane. Moreover, most SDMs induced in response to DS were classified as lipids, others (sugars and alcohols), amino acids and derivatives, and alkaloids. KEGG analysis showed that 2-oxocarboxylic acid metabolism (13 up-regulated and 5 down regulated SDMs), ABC transporters (29 up-regulated and 1 down regulated SDMs), biosynthesis of amino acids (19 up-regulated and 7 down regulated SDMs), glucosinolate biosynthesis (8 up-regulated SDMs), and valine, leucine, and isoleucine biosynthesis (4 up-regulated and 3 down regulated SDMs) were the top-5 KEGG enriched pathways. There were 2 KEGG enriched pathways in M_DS/COS, namely purine metabolism (4 up-regulated and 5 down regulated SDMs) and phenylalanine metabolism (1 up-regulated and 5 down-regulated SDMs). Thus, the modulation of DS by chitooligosaccharide in sugarcane was mainly related to phenylalanine metabolism.

Through integrative analysis of the transcriptome and metabolome, the strong red hue of red pericarp longan is due to accumulation of cyanidin derivatives in the pericarp [30]. The genes F3′H and F3′5′H may play an important role in selecting which components of anthocyanins will be synthesized [30]. Therefore, combined transcriptome and metabolome analysis can also reveal many important metabolic processes that have powerful effects. To identify the molecular regulatory network of genes and metabolites of sugarcane under DS, as well as the regulation by chitooligosaccharide, we carried out an integrative analysis between the transcriptome and metabolome. The integrative analysis revealed 8 common KEGG enriched pathways in both T_CG/DS and M_CG/DS, of which pyruvate metabolism was closely related to photosynthesis and respiration. Meanwhile, pyruvate is a hub for the mutual conversion of sugars, fats, and amino acids [31]. In this study, only D-lactic acid levels were up-regulated and other SDMs (L-malic acid, pyruvic acid, 2-propylmalic acid, and 2-isopropylmalic acid) in the pyruvate metabolism were down-regulated, indicating that respiration in sugarcane was restrained under DS. Pyruvate was closely related to 14 DEGs (|r| > 0.99), of which only 1 DEG, the pyruvate kinase *Sspon.02G0043670-1B*, had a positive correlation with pyruvate. Pyruvate kinases are up-regulated by abiotic stress and hormones that can be involved in plant stress defenses [32]. In rice, the pyruvate kinase *OsPK1* significantly regulates monosaccharide metabolism, sucrose transport, and GA/ABA balance [33], whereas *OsPK2* regulates endosperm development and grain filling [34,35]. Therefore, *Sspon.02G0043670-1B* could be an important regulatory gene that participates in the regulation of drought resistance of sugarcane and may be a key regulatory gene for sugarcane growth and development. Other genes showed a significant correlation with D-lactate and malate, including *Sspon.02G0026610-2B*, *Sspon.03G0006680-1A*, *Sspon.04G0012960-2B*, *novel.25810*, *Sspon.05G0008290-3C*, *Sspon.03G0006680-1P*, and *Sspon.02G0013900-1A*, which are regulatory genes of pyruvate metabolism under DS. Further research is needed to characterize in detail the role of these genes in drought stress responses.

The results of the integrative analysis between the transcriptome and metabolome further showed that phenylalanine metabolism was the only common KEGG-enriched pathway between T_DS/COS and M_DS/COS. Phenylalanine is a central amino acid in plants and is the precursor for many key secondary metabolites, such as lignin, phenylpropanoids, and flavonoids, that are involved in various biotic and abiotic stresses [36,37]. Additional study of the regulation of phenylalanine metabolism by chitooligosaccharide in sugarcane under DS would be valuable, particularly since 13 DEGs (7 up-regulated and 6 down-regulated) and 6 SDMs (1 up-regulated and 5 down-regulated) that are associated with phenylalanine metabolism were found in the T_DS/COS and M_DS/COS, respectively. Phenylalanine ammonia-lyase (PAL) is a crucial enzyme that has roles ranging from primary metabolism to secondary phenylpropanoid metabolism in plants, and is critical for plant growth, development, and stress defense. In potato, expression levels of *StPAL1*, *StPAL6*, *StPAL8*, *StPAL12*, and *StPAL13* were significantly up-regulated under drought and high temperature stress, indicating that they may be involved in defense against high temperature and DS [38]. Cinnamic acid is an organic acid that occurs naturally in plants and has a broad spectrum of biological activities with low toxicity [39]. In this study, we found that 5 PAL genes and levels of cinnamic acid were up-regulated while phenylalanine was down-regulated, suggesting that the chitooligosaccharide positively regulated phenylalanine metabolism under DS in sugarcane. The 5 PAL genes (*Sspon.04G0008040-5P*, *Sspon.04G0008040-7P*, *Sspon.04G0008040-9P*, *Sspon.04G0008040-10P*, and *novel.31257*) may be candidate genes for drought resistance in sugarcane. Indeed, a previous study showed that chitooligosaccharide could enhance drought resistance of wheat seedings, resulting in the promotion of photosynthesis and carbohydrate metabolism [40]. The physiological data in the present study also showed that chitooligosaccharide plays a regulatory role in increasing chlorophyll content and improving photosynthesis under DS. Taken together, our results show that chitooligosaccharide can improve drought resistance of sugarcane. The results further suggest that for cultivation of sugarcane, chitooligosaccharide (50 mg/L) sprayed on foliage at 450 L/hm^2^ at jointing stage can improve sugarcane yield and quality.

## 4. Materials and Methods

### 4.1. Plant Materials and Drought Stress

The drought-tolerant cultivar ROC22 (*Saccharum* spp. hybrid) used in this study was provided by the National Engineering Research Center for Sugarcane, Fujian Agriculture, and Forestry University. Sugarcane stems were cut into single-bud segments that were soaked in 0.5% carbendazim solution for 24 h before placement in pots (12 cm in diameter and height) containing pine needle soil. The pots were incubated at 30 °C in a constant temperature incubator. When the seedlings reached the 2-leaf stage, 3 seedlings for each treatment having consistent growth potential were transferred to a bucket (45 cm diameter, 30 cm height) with 35 kg mixed soil (clay soil:pine needle soil = 3:1 (*v*:*v*)). A total of 9 buckets, labeled 1–9, were placed in a greenhouse and watered every 2 days. When the plants reached the 8-leaf stage, plants in buckets 1–3 (control group, CG) were watered as usual, whereas those in buckets 4–6 (drought stress group, DS) received no additional watering. Plants in buckets 7–9 (DS + COS (≤3200 Da), COS) also were not watered but were sprayed with 50 mg/L COS solution at a dose of 15 mL/bucket. At 9:00 pm on day 10 of exposure to DS, the photosynthetic activity of the +1 and −1 leaves was measured with a portable photosynthesis system (LI6400, America) and chlorophyll content of the +1 and −1 leaves was measured with a chlorophyll meter (SPAD-502PLUS, Japan). At the same time, the +1 and −1 leaves of plants in each group were clipped and placed immediately in a Ziplock bag before freezing in liquid nitrogen. These leaf samples were stored in −80 ℃ refrigerator and used for experiments of physiology, transcriptome, RT-qPCR, and metabolome.

### 4.2. Measurement of Physiological Parameters

Free proline and soluble sugar concentrations were determined using the acid ninhydrin reagent method and the anthrone method, respectively [41]. Malondialdehyde (MDA) concentration was determined using the thiobarbituric acid method [42].

### 4.3. Rna Sequencing and Data Analysis

Nine samples of total RNA were extracted using NA Isolater Total RNA Extraction Reagent according to the manufacturer’s instructions (Novizan Biotechnology Co., LTD, Nanjing, China). The samples were termed T_CG, T_DS, and T_COS, respectively. The cDNA library was constructed and sequenced on an Illumina HiSeq X-ten platform at the Wuhan Metware Biotechnology Co., Ltd. (Wuhan, China) and the length of generated reads was 150 bp paired-end (PE 150). To obtain clear reads, low-quality reads, adapter sequences, and sequences having > 10% poly-N in the raw reads were filtered. The Q20, Q30 and GC contents of each sample were calculated. Clear reads from every sample were mapped to the *Saccharum spontaneum* [43] reference genome using HISAT2 [44]. Raw counts of genes were determined using feature counts [45]. Differentially expressed genes (DEGs) between two samples were identified using DESeq2 with |log_2_^fold change^| ≥ 1 and a false discovery rate (FDR) < 0.05 [46]. The function of DEGs was annotated using the KEGG and GO databases. KEGG pathway analysis of DEGs was performed with BLAST software [47] and KEGG enrichment was analyzed using KOBAS 2.0 software with *p*-value < 0.05 [48,49]. GO analysis of DEGs was carried out using the R package cluserProfiler [50]. Transcription factors (TFs) among the DEGs were predicted using iTAK [51] software with PlnTFDB [52] and PlantTFDB [53] databases.

### 4.4. Real-Time Quantitative Pcr (Rt-qpcr)

Ten candidate DEGs were verified by RT-qPCR to evaluate the accuracy of RNA-Seq. Based on the coding gene sequences, RT-qPCR primers were designed using primer premier 6.0 software. Glyceraldehyde-3-phosphate dehydrogenase (*GAPDH*) was selected as the internal control gene (Table S13). The SYBR^®®^ Green Premix Pro Taq HS qPCR Kit (Novizan Biotechnology Co., LTD, Nanjing, China) was used for the RT-qPCR assay using 20 μL reaction solutions containing that 10 μL 2 × ChamQ Universal SYBR qPCR Master Mix, 0.5 μL primer F (10 μM), 0.5 μL primer R (10 μM), 1 μL cDNA and 8 μL nuclease-free water. The qPCR reactions involved denaturation at 95 °C for 30 s, followed by 40 cycles of 5 s at 95 °C and 30 s at 60 °C. The RT-qPCR assays were carried out using a QuantStudio^®®^ Real-Time PCR system (Applied Biosystems, Foster City, CA, USA). The qPCR data were analyzed using the 2^−ΔΔCt^ quantitative method to determine differences in gene expression [54]. Three independent biological replicates and three technological replicates were used for each sample in this study.

### 4.5. Widely Targeted Metabolomics and Data Analysis

For metabolomic analysis, there were three biological replicates for each group termed M_CG (control group), M_DS (drought stress group), M_COS (drought stress + chitooligosaccharide group). Biological samples were freeze-dried in a vacuum freeze-dryer (Scientz-100F) and then crushed using a mixer mill (MM 400, Retsch) with a zirconia bead for 1.5 min at 30 Hz. Then, 100 mg of lyophilized powder was dissolved in 1.2 mL 70% methanol solution, vortexed for 30 s every 30 min for 6 times total, and then incubated at 4 °C overnight. Following centrifugation at 12,000 rpm for 10 min, the extracts were filtered (SCAA-104, 0.22μm pore size; ANPEL, Shanghai, China) before use in UPLC-MS/MS analysis. 

Results for hierarchical cluster analysis (HCA) of samples and metabolites are presented as heatmaps with dendrograms. Pearson correlation coefficients (PCC) between samples were calculated using the core function in R and presented as only heatmaps. Both HCA and PCC were carried out using the R package heatmap. For HCA, normalized signal intensities of metabolites (unit variance scaling) are visualized as a color spectrum. Principal component analysis (PCA) was performed with the statistics function prcomp within R (www.r-project.org, accessed on 10 July 2022). The data was unit variance scaled before PCA. Significantly different metabolites (SDMs) between groups were determined by VIP ≥ 1 and absolute Log_2_FC (fold change) ≥ 1. VIP values were extracted from OPLS-DA results, which also contained score plots and permutation plots, and was generated using R package MetaboAnalystR. The data was log transformed (log_2_) and mean centering before OPLS-DA. To avoid overfitting, a permutation test (200 permutations) was performed. Identified metabolites were annotated using the KEGG compound database (http://www.kegg.jp/kegg/compound/, accessed on 10 July 2022), and annotated metabolites were then mapped to the KEGG pathway database (http://www.kegg.jp/kegg/pathway.html, accessed on 10 July 2022). Pathways with the SDMs mapped were then fed into MSEA (metabolite sets enrichment analysis), and their significance was determined using *p*-values hypergeometric test. 

### 4.6. UPLC Conditions

The sample extracts were analyzed using an UPLC-ESI-MS/MS system (UPLC, SHIMADZU Nexera X2; MS, Applied Biosystems 4500 Q TRAP) with an Agilent SB-C18 (1.8 µm, 2.1 mm × 100 mm) column. The mobile phase consisted of solvent A, pure water with 0.1% formic acid, and solvent B, acetonitrile with 0.1% formic acid. Sample measurements were performed with a gradient program starting with 95% A, 5% B, followed by a linear gradient of 5% A to 95% B over 9 min, and then holding at this composition for 1 min. Subsequently, a composition of 95% A, 5.0% B was reached within 1.1 min and maintained for 2.9 min. The flow was 0.35 mL/min and the column oven was set to 40 °C with a 4 μL injection volume. The effluent was alternatively connected to an ESI-triple quadrupole-linear ion trap (QTRAP)-MS.

### 4.7. ESI-Q TRAP-MS/MS

LIT and triple quadrupole (QQQ) scans were acquired on a triple quadrupole-linear ion trap mass spectrometer (Q TRAP), AB4500 Q TRAP UPLC/MS/MS System, equipped with an ESI Turbo Ion-Spray interface, operating in positive and negative ion mode and controlled by Analyst 1.6.3 software (AB Sciex). The ESI source operation parameters were: ion source, turbo spray; source temperature, 550 °C; and ion spray voltage (IS) 5500 V (positive ion mode)/−4500 V (negative ion mode). The ion source gas I (GSI), gas II(GSII), and curtain gas (CUR) were set at 50, 60, and 25 psi, respectively. The collision-activated dissociation (CAD) was high. Instrument tuning and mass calibration were performed with 10 and 100 μM polypropylene glycol solutions in QQQ and LIT modes, respectively. QQQ scans were acquired as MRM experiments with collision gas (nitrogen) set to medium. DP and CE for individual MRM transitions were measured with further DP and CE optimization. A specific set of MRM transitions was monitored for each period according to the metabolites that were eluted during this period.

### 4.8. Statistical Analysis

Duncan’s multiple comparison method was conducted using Statistical Product and Service Solutions software (IBM SPSS 19.0) to assess the differences in physiological indices among the three different treatments (*p* < 0.05). The bar diagram was drawn using Microsoft Office Excel 2016. Pearson correlation analysis of gene–metabolites was performed using SPSS 19.0 (threshold for association analysis > 0.90, *p* < 0.01). Gene–metabolite correlation network diagrams were visualized using Cytoscape software [55].

## 5. Conclusions

In this study, physiology analyses showed photosynthesis and physiological activity of sugarcane plants were inhibited under drought stress (DS), and that chitooligosaccharide treatment could alleviate DS to a certain extent. Numerous differentially expressed genes (DEGs) and significantly different metabolites (SDMs) related to DS were identified through transcriptome analysis and metabolome analysis. For DS/COS, most DEGs were up-regulated and most SDMs were down-regulated relative to CG/DS, demonstrating that chitooligosaccharide could alleviate the negative effects of DS in sugarcane. Combined transcriptome and metabolome analyses revealed eight common KEGG-enriched pathways in the CG/DS group, of which pyruvate metabolism was the most closely related to sugarcane growth and development. These findings provide the basis for further research into the role of pyruvate metabolism in drought stress tolerance of sugarcane. Chitooligosaccharide positively regulated phenylalanine metabolism of sugarcane under DS, with induction of synthesis of additional downstream phenylalanine secondary metabolites that can participate in metabolic activities in response to DS.

## Data Availability

Raw sequence data for RNA-sequencing were deposited in the NCBI database under Bioproject number PRJNA866695. Metabolite data were deposited in OMIX (https://ngdc.cncb.ac.cn/omix, accessed on 10 July 2022: accession no. OMIX001521) at the China National Center for Bioinformation/Beijing Institute of Genomics, Chinese Academy of Sciences.

## Supplementary Materials

The following supporting information can be downloaded at: https://www.mdpi.com/article/10.3390/ijms23179737/s1.
