# Supplementary material for: Integrated Analysis of Transcriptome and Metabolome Reveals the Regulation of Chitooligosaccharide on Drought Tolerance in Sugarcane (Saccharum spp. Hybrid) under Drought Stress
Source: Int J Mol Sci. 2022 Aug 27;23(17):9737. doi: 10.3390/ijms23179737 (PMC9456405; doi:10.3390/ijms23179737)
Supplement: Supplementary file 1 [file ijms-23-09737-s001.zip › Supplementary figures.pdf]

Integrated analysis of transcriptome and metabolome reveals the regulation of chitooligosaccharide on drought tolerance in sugarcane (*Saccharum* spp. hybrid) under drought stress

Shan Yang[1], Na Chu[2], Hongkai Zhou[1], Jiashuo Li[1], Naijie Feng[1], Junbo Su[3], Zuhu Deng[2], Xuefeng Shen[1]*, Dianfeng Zheng[1]*

[1]College of Coastal Agricultural Sciences, South China Branch of National Saline-Alkali Tolerant Rice Technology Innovation Center, Guangdong Ocean University, Zhanjiang, China, 524088

[2]National Engineering Research Center for Sugarcane, Fujian Agriculture and Forestry University, Fuzhou, China, 350002

[3]South Subtropical Crops Research Institute, Chinese Academy of Tropical Agricultural Science, Zhanjiang, China, 524091

***Correspondence**

Xuefeng Shen

shenxuefeng@gdou.edu.cn

Dianfeng Zheng

zhengdf@gdou.edu.cn

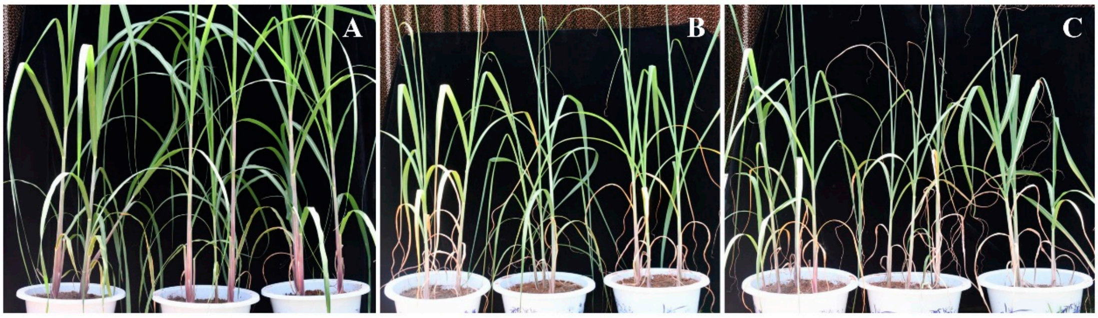

Figure S1. Phenotype of ROC22 exposed to three different treatments. A: control group (CG). B: drought stress (DS). C: drought stress + chitooligosaccharide (COS).

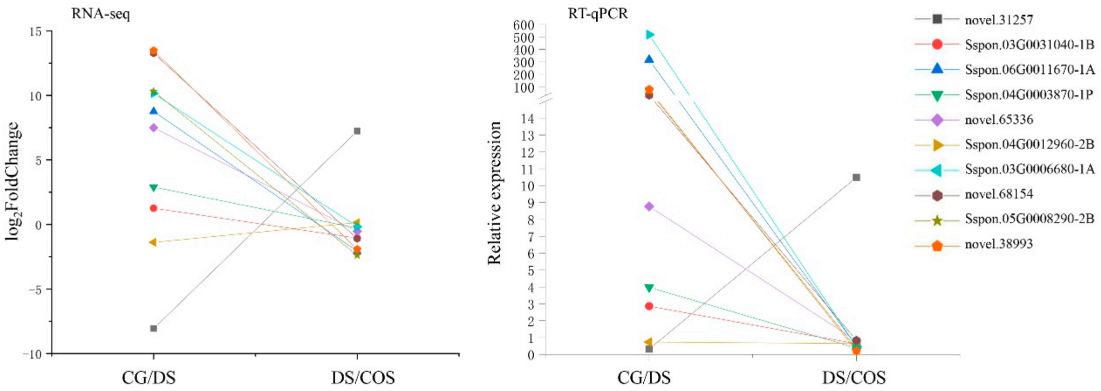

Figure S2. RT-qPCR and RNA-seq results for 10 genes in the two groups.

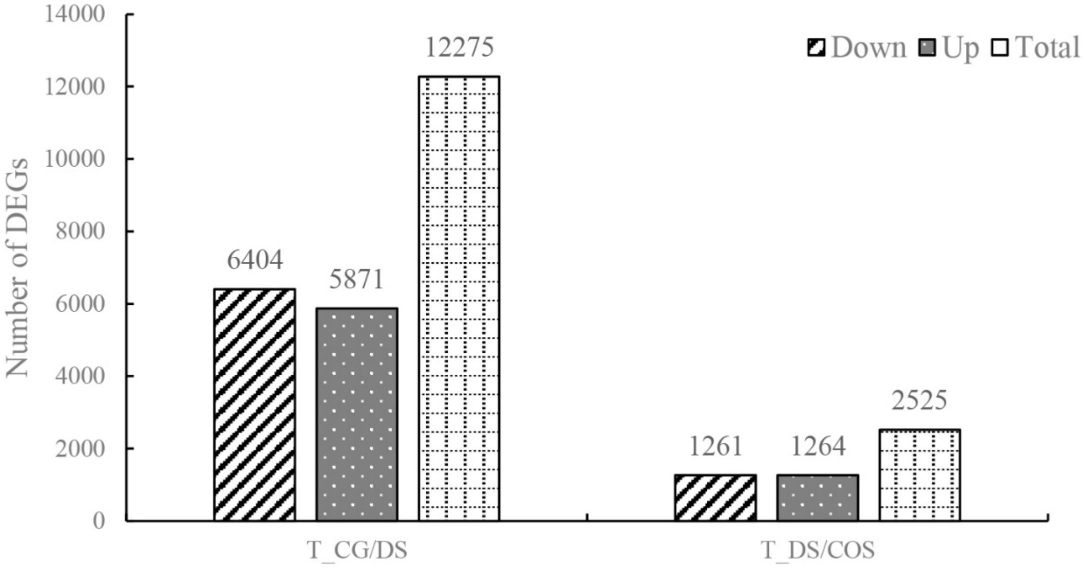

Figure S3. Number of DEGs.

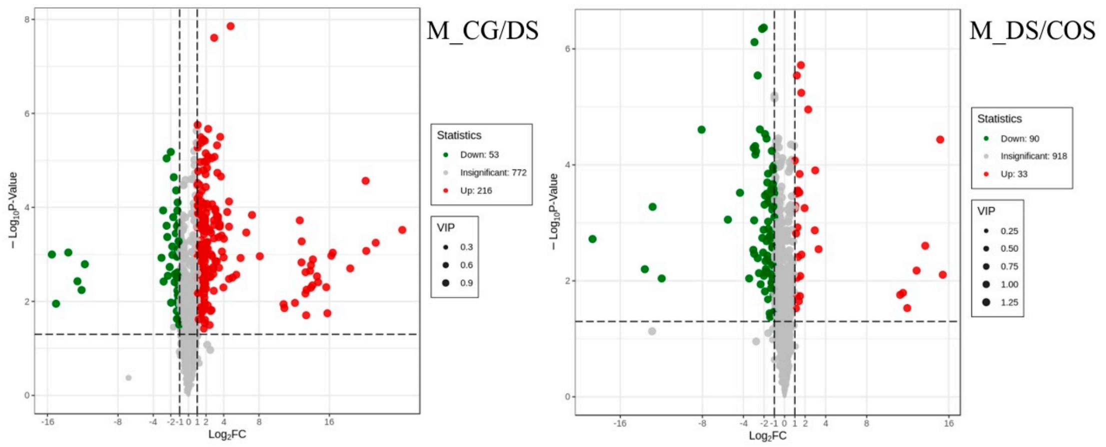

Figure S4. Volcano plot of SDMs.

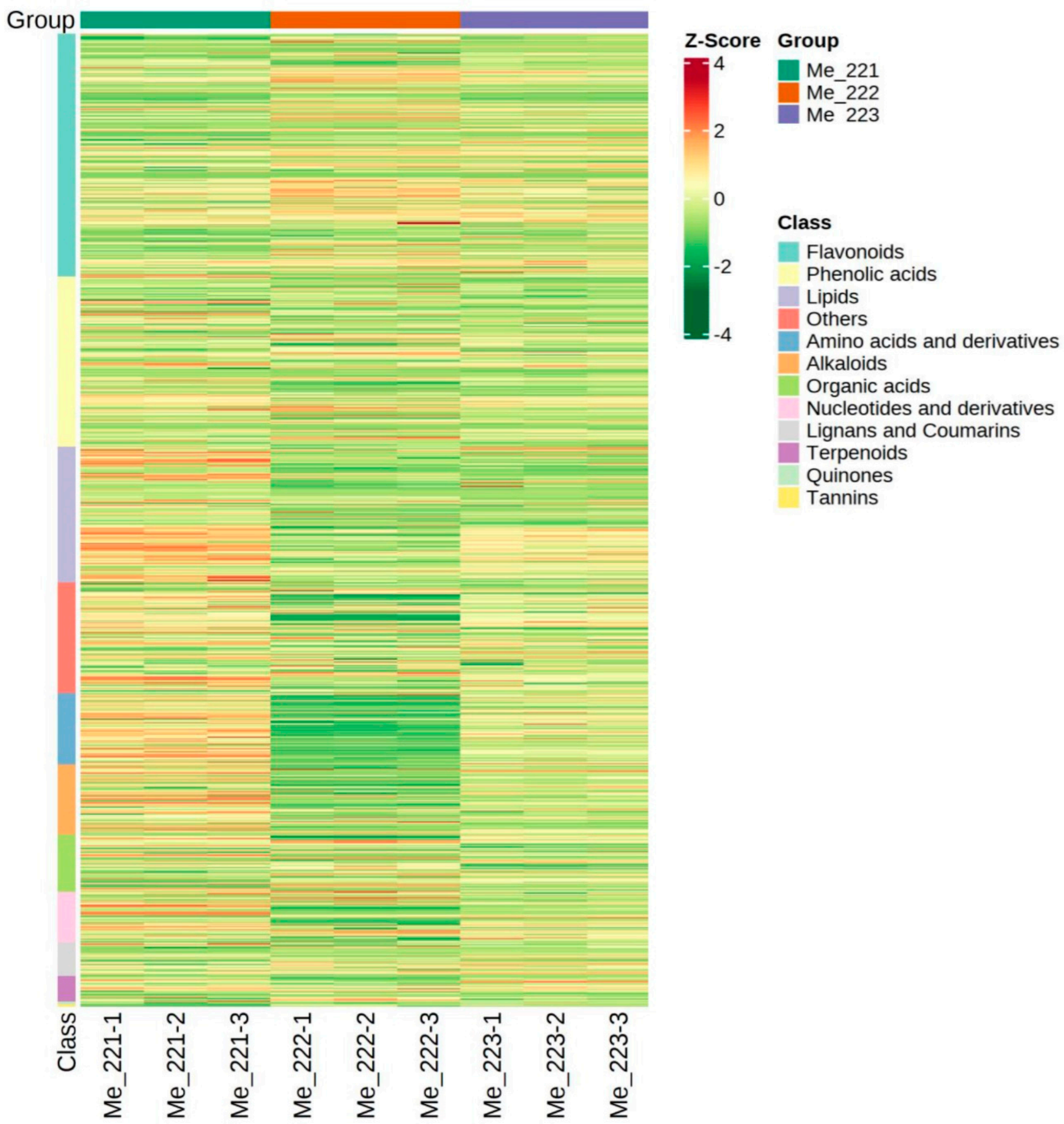

Figure S5. Heatmap hierarchical clustering of all detected metabolites.

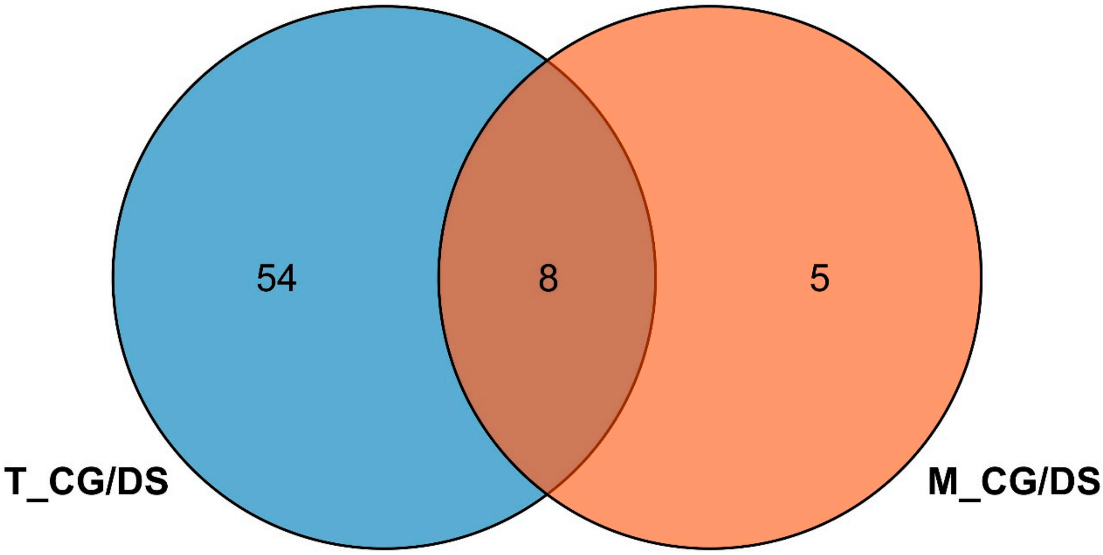

Figure S6. Venn diagram between T_CG/DS and M_CG/DS.
