# Supplementary material for: Integrated Analysis of Transcriptome and Metabolome Reveals the Regulation of Chitooligosaccharide on Drought Tolerance in Sugarcane (Saccharum spp. Hybrid) under Drought Stress
Source: Int J Mol Sci. 2022 Aug 27;23(17):9737. doi: 10.3390/ijms23179737 (PMC9456405; doi:10.3390/ijms23179737)
Supplement: Supplementary file 1 [file ijms-23-09737-s001.zip › Supplementary table legends.pdf]

Integrated analysis of transcriptome and metabolome reveals the regulation of chitooligosaccharide on drought tolerance in sugarcane (*Saccharum* spp. hybrid) under drought stress

Shan Yang[1], Na Chu[2], Hongkai Zhou[1], Jiashuo Li[1], Naijie Feng[1], Junbo Su[3], Zuhu Deng[2], Xuefeng Shen[1]*, Dianfeng Zheng[1]*

[1]College of Coastal Agricultural Sciences, South China Branch of National Saline-Alkali Tolerant Rice Technology Innovation Center, Guangdong Ocean University, Zhanjiang, China, 524088

[2]National Engineering Research Center for Sugarcane, Fujian Agriculture and Forestry University, Fuzhou, China, 350002

[3]South Subtropical Crops Research Institute, Chinese Academy of Tropical Agricultural Science, Zhanjiang, China, 524091

***Correspondence**

Xuefeng Shen

shenxuefeng@gdou.edu.cn

Dianfeng Zheng

zhengdf@gdou.edu.cn

Supplementary table legends

Table S1. The quality of RNA-seq data.

Table S2. GO enrichment in T_CG/DS.

Table S3. GO enrichment in T_DS/COS.

Table S4. Significantly enriched KEGG pathways for DEGs in T_CG/DS.

Table S5. Significantly enriched KEGG pathways for DEGs in T_DS/COS.

Table S6. Classification of SDMs.

Table S7. Up- and down- regulated SDMs of lipids, others, amino acids and derivatives and alkaloids in CG/DS.

Table S8. Up- and down- regulated SDMs of lipids, others, amino acids and derivatives and alkaloids in DS/COS.

Table S9. Top-20 enriched KEGG pathways for SDMs in M_CG/DS.

Table S10. Top-20 enriched KEGG pathways for SDMs in M_DS/COS.

Table S11. Correlation of DEGs and SDMs in pyruvate metabolism in the CG/DS group.

Table S12. Correlation of DEGs and SDMs in phenylalanine metabolism in the DS/COS group.

Table S13. RT-qPCR primers.
